# A Novel Smoking Cessation Smartphone App Integrated With a Mobile Carbon Monoxide Checker for Smoking Cessation Treatment: Protocol for a Randomized Controlled Trial

**DOI:** 10.2196/12252

**Published:** 2019-02-11

**Authors:** Akihiro Nomura, Hiroki Tateno, Katsunori Masaki, Tomoyasu Muto, Shin Suzuki, Kohta Satake, Eisuke Hida, Koichi Fukunaga

**Affiliations:** 1 CureApp Institute Karuizawa Japan; 2 Innovative Clinical Research Center Kanazawa University Kanazawa Japan; 3 Department of Cardiovascular and Internal Medicine Kanazawa University Graduate School of Medicine Kanazawa Japan; 4 Division of Pulmonary Medicine Department of Medicine Keio University School of Medicine Tokyo Japan; 5 Department of Internal Medicine Saitama City Hospital Saitama Japan; 6 CureApp, Inc Tokyo Japan; 7 Department of Biostatistics and Data Science Osaka University Graduate School of Medicine Osaka Japan

**Keywords:** continuous abstinence rate, nicotine dependence, telemedicine, randomized controlled trial, smoking cessation, tobacco use disorder

## Abstract

**Background:**

Smoking cessation treatment programs have been widely available for patients with nicotine dependence. Despite intensive programs, the continuous abstinence rate (CAR) from weeks 9-12 is still about 50%. Recently, a smartphone app emerged as a novel tool for therapeutic interventions, including nicotine dependence. In this study, we developed “CureApp Smoking Cessation” (CASC), which consists of a smartphone app for patients and a Web-based patient management software for doctors with a mobile carbon monoxide (CO) checking device to improve the efficacy of the smoking cessation treatment.

**Objective:**

This study aims to evaluate whether the CASC app is effective for individuals with nicotine dependence in addition to standard smoking cessation programs.

**Methods:**

This will be a randomized, sham-controlled, open-label, multicenter trial. We will recruit participants with nicotine dependence, but are otherwise healthy adults. We will randomize and allocate participants 1:1 to the CASC treatment group or a control app group. Both groups will receive a 12-week standard smoking cessation program with pharmacotherapy and counseling. In addition, participants in the treatment group will have the CASC app installed on their smartphone, which will provide video tutorials, advice from an artificial intelligence nurse, a digital diary, and measure daily exhaled CO concentration. In contrast, the control group will have the control app installed on their smartphone, where all the functions that can potentially effect smoking cessation are removed. The primary outcome will be the biochemically validated CAR from weeks 9-24. The success of smoking cessation will be defined as self-reported continuous abstinence from weeks 9-24 and exhaled CO concentration ≤10 ppm both at weeks 12 and 24. The main secondary outcomes will be the CAR from weeks 9-12, weeks 9-52, and 7-day point prevalence abstinence at weeks 4, 8, 12, 24, and 52.

**Results:**

We will recruit 580 participants with nicotine dependence from October 2017 to September 2018 or until the recruitment process is complete. The final 52-week follow-up will be completed in October 2019. We expect all trial results to be available by the end of 2019. The trial is funded by CureApp, Inc.

**Conclusions:**

This is the first randomized controlled trial to evaluate the efficacy of CASC. We expect that CASC, in addition to standard smoking cessation programs, has a significantly higher CAR during weeks 9-24 than the control app.

**Trial Registration:**

University Hospital Medical Information Network Clinical Trials Registry UMIN000031589; https://upload.umin.ac.jp/cgi-open-bin/ctr/ctr_view.cgi?recptno=R000033555

**International Registered Report Identifier (IRRID):**

DERR1-10.2196/12252

## Introduction

Smoking is a risk factor that causes various diseases such as malignant tumors, heart disease, cerebrovascular disease, and chronic obstructive pulmonary disease [1]. It has been estimated that there are 22 million (approximately 18% of the total Japanese population) of adult smokers in Japan, and the number of deaths attributable to smoking is approximately 129,000 per year, indicating that smoking is the most common extrinsic cause of death among noninfectious diseases [2]. In addition, excess medical expenses owing to smoking are up to 1.5 trillion yen (US $13 billion) [3]. Therefore, reducing smoking prevalence could contribute to not only prevent the onset of these lifestyle-related diseases and cancer but also decrease future medical costs [4].

Smoking cessation treatment programs have been widely available for patients with nicotine dependence supported by the Japanese national insurance system [5]. This program consists of 5 visits spanning 12 weeks providing counseling by health care professionals and pharmacotherapy, including nicotine patch and varenicline. Despite this intensive treatment program, the continuous abstinence rate (CAR) from weeks 9-12 has still been 40%-65% [6-8]. Moreover, it significantly decreases after the program finishes and is around 40% at 1-year follow-up [7]. To improve both in-program and long-term CAR, we need additional and more effective treatment interventions both during the regular outpatient clinic visits and after the completion of the 12-week program [6].

Medical apps for smartphones are emerging as “therapeutic” intervention tools to ameliorate diabetes mellitus [9,10], depression [11], and nicotine dependence [12,13]. Because they cover interoutpatient visit blank periods (ie, intervals between clinic visits during which patients normally cannot obtain any clinical guidance from medical staffs), these apps are useful to continuously monitor, promote, and encourage the treatment program from medical staff, as well as by patients themselves. In this regard, Free et al [13] reported that smoking cessation support with daily or weekly text messages during the program doubled smoking quit rates. Although smartphone apps for nicotine dependence are already available in the United States, it remains unclear whether the app with a mobile carbon monoxide (CO) checker device is safe and effective in patients with nicotine dependence.

Recently, we developed “CureApp Smoking Cessation” (CASC), which consists of the latest version of CureApp Smoking Cessation smartphone app for patients and a Web-based patient management software for primary physicians with a mobile CO checking device to improve the success of the smoking cessation treatment. The CASC provides users with accurate knowledge of nicotine dependence, clues to change their behaviors, and measurements of their own exhaled CO concentration levels at home. In addition, patients can share these data remotely with their primary physicians. Masaki et al reported that, in the previous prospective studies with participants using prototypes of CASC smartphone app without a mobile CO checker, the results showed the feasibility and usability in the phase I study [14] and demonstrated a higher CAR from weeks 9­-24 than the national surveys in the phase II study [15].

Therefore, this study aims to evaluate whether the latest version of CASC in addition to a standard smoking cessation program is effective in treating individuals with nicotine dependence.

## Methods

### Overall Trial Design

**Figure 1:**
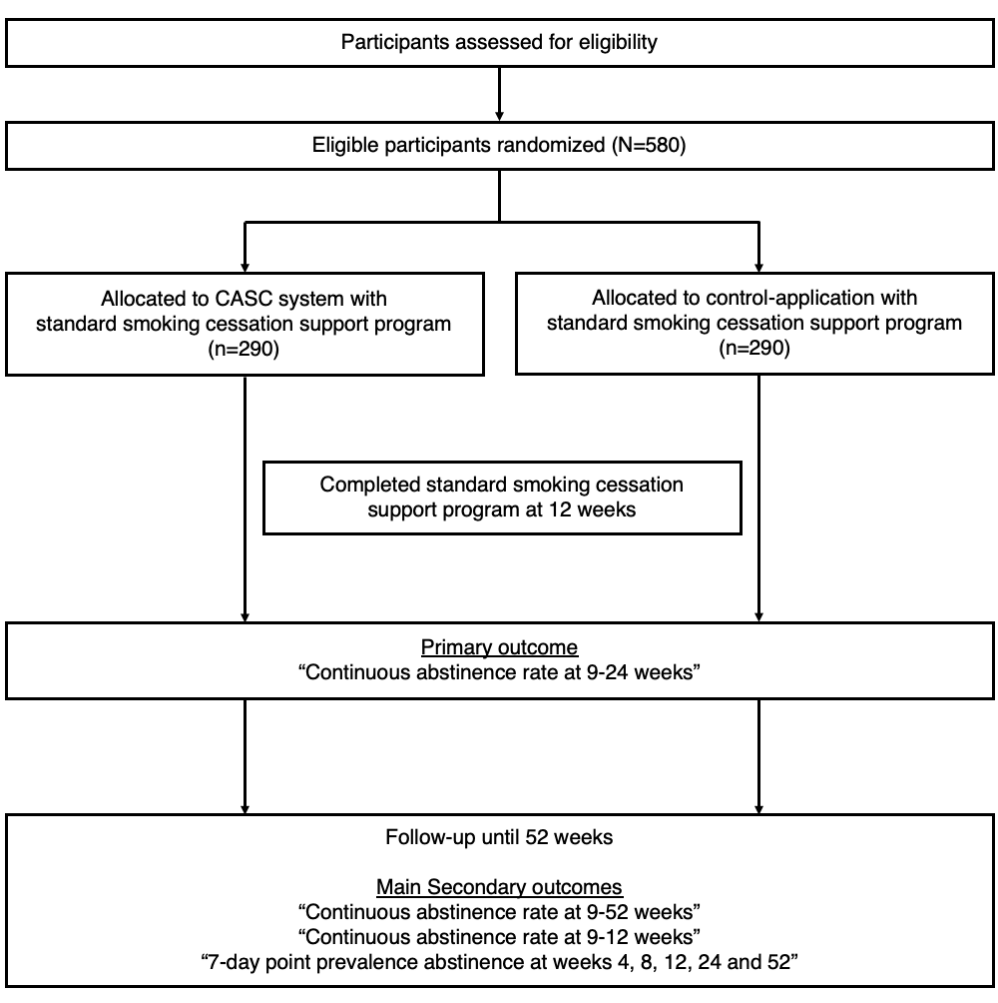
Flowchart of the trial protocol. CASC: CureApp Smoking Cessation.

Figure 1 shows the flowchart of the trial. This trial will be a randomized, sham-controlled, open-label, multicenter trial. Table 1 shows trial centers. This trial aims to assess the efficacy of CASC in patients with nicotine dependence when added to a standard smoking cessation program. The treatment group will use the CASC smartphone app integrated with a mobile CO checker and a Web-based patient management software for 24 weeks. Table 2 shows the overall follow-up schedule. The primary outcome of this study will be the CAR from weeks 9-24. We hypothesize that the CASC treatment group will have a higher CAR than the control app group from weeks 9-24. The CAR is defined as the percentage of individuals continuously not smoking even a puff during the specified period. We will conduct this trial in compliance with the Declaration of Helsinki, Medical Device Good Clinical Practice, Ethical Guidelines for Medical and Health Research Involving Human Subjects, and all other applicable laws and guidelines in Japan. This protocol and informed consent forms were approved by the Institutional Review Board at Keio University School of Medicine and affiliated institutions. Furthermore, we will use the latest version of the approved documents. This study is registered at the University Hospital Medical Information Network Clinical Trials Registry (UMIN000031589).

### Trial Participants

We will recruit nicotine-dependent adult participants from October 2017 to September 2018 or until the recruitment process completes; we plan to follow them until October 2019. Participants who meet all inclusion criteria will be included in the trial and those fulfilling the exclusion criteria will be removed from this trial (Textbox 1). We will obtain written informed consent from all trial participants. Participants should understand the content of the consent form sufficiently before their acceptance*.* Consent forms must be dated and signed both by trial participants and researchers. When consent is obtained, the first copy of the form will be kept in each institution, while the other part will be kept by participants and will not be collected even after the trial is over. In addition, we will inform all participants that their medical care will not be affected if they refuse to enroll in this trial. Participants will be able to leave the trial whenever they want to discontinue it. All participants will receive a compensation of 10,000 yen per clinic visit (7 visits in total).

### Randomization

Randomization will be performed for eligible participants. They will be allocated 1:1 to both arms—the CASC treatment group and the control app group. Randomization will be performed by a computer-generated random sequence with stratification for smoking cessation medications.

**Table 1:** The list of trial centers.

Number	Institution	Prefecture
1	Ankou Medical Clinic	Tokyo
2	Ayano Clinic	Saitama
3	BOOCS Clinic Fukuoka	Fukuoka
4	Chuo Naika Clinic	Tokyo
5	Ebisu Garden Place Clinic	Tokyo
6	Higashi-hie Naika	Fukuoka
7	Hosoda Shinryojo (clinic)	Tokyo
8	Inoue Naika Clinic	Ibaraki
9	Kanda Clinic	Tokyo
10	Keio University Hospital	Tokyo
11	Kimura-Shiro Clinic	Fukuoka
12	Kita Shin-Yokohama Naika Clinic	Kanagawa
13	Mashiba Iin (clinic)	Saitama
14	Maekawa Medical Clinic	Kanagawa
15	Miyazaki RC Clinic	Tokyo
16	Motosumiyoshi Kokoromi Clinic	Kanagawa
17	Nakameguro Atlas Clinic	Tokyo
18	National Center for Global Health and Medicine	Tokyo
19	Nemoto Geka-Seikeigeka (clinic)	Saitama
20	Nihonbashi Naika-Allegy Clinic	Tokyo
21	Nomura Iin (clinic)	Tokyo
22	Odayaka Life Naika Clinic	Saitama
23	Saitama City Hospital	Saitama
24	Sawayama Naika-Sougoushinryou Clinic	Fukuoka
25	Segawa Hospital	Saitama
26	Shimizu Clinic Fusa	Saitama
27	Shinjuku Research Park Clinic	Tokyo
28	Sone Clinic Shinjuku	Tokyo
29	Tajima Clinic	Tokyo
30	Tenjin Sogo Clinic	Fukuoka
31	Ueda Naika Clinic	Fukuoka

### Intervention and Control

In addition to the 12-week standard treatment procedure for smoking cessation [5], we will prescribe the CASC to the treatment group. On the other hand, a control app will be prescribed to the control group. The standard 12-week smoking cessation treatment protocol provides 5 on-site examinations and in-person counseling by a primary physician at each outpatient clinic within 3 months.

**Figure 2:**
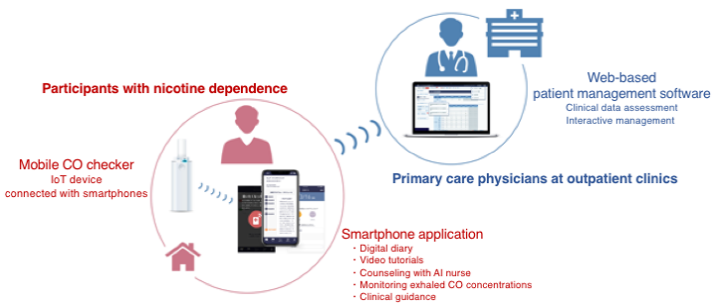
Overview of the "CureApp Smoking Cessation". CO: carbon monoxide; IoT: Internet of Things.

CASC consists of a CureApp Smoking Cessation smartphone app based on a cloud system paired with a mobile CO checker for participants and a Web-based patient management software for primary care physicians. Participants in the treatment group will install the app in their smartphones and then begin taking a few minutes every day for watching a video tutorial regarding smoking cessation; in addition, participants from the treatment group will keep a digital diary of quitting smoking, chat with artificial intelligence (AI) nurse, and check their exhaled CO concentration by the mobile CO checker at least once a day. Physicians will be able to follow participants’ exhaled CO concentration data and physical conditions through a secure cloud system and review them during the clinic visits (Figure 2).

**Table 2:** The trail assessment and evaluation schedule.

Assessments	Observational period							At withdrawal
	Day of registration (Day 1)	2 weeks (Day 15)	4 weeks (Day 29)	8 weeks (Day 57)	12 weeks (Day 85)	24 weeks (Day 169)	52 weeks (Day 366)	
Patients’ background	✓							
Tobacco Dependence Screener	✓							
Brinkman Index^a^	✓							
Fagaström Test for Nicotine Dependence	✓							
12-item French version of the Tobacco Craving Questionnaire	✓	✓	✓	✓	✓	✓	✓	
Kano Test for Social Nicotine Dependence	✓			✓	✓	✓	✓	
Mood and Physical Symptoms Scale	✓	✓	✓	✓	✓	✓	✓	
Exhaled carbon monoxide concentration	✓	✓	✓	✓	✓	✓	✓	✓
Status of nonsmoking	✓	✓	✓	✓	✓	✓	✓	✓
Status of device use		✓	✓	✓	✓	✓		✓
Adverse events	✓	✓	✓	✓	✓	✓	✓	✓

^a^Brinkman index=(number of cigarettes per day)×(number of smoking years).

**Inclusion and exclusion criteria.**

Inclusion:

- Participants diagnosed with nicotine dependence (Tobacco Dependence Screener≥5 points)
- Participants with Brinkman index≥200
- Participants who wish to quit smoking immediately
- Participants who agree to receive the smoking cessation treatment program and sign the written consent form
- Participants who can use a smartphone (operating system: Android 5.0 and above or iPhone 8.0 and above)

Exclusion:

- Participants with severe mental illness
- Participants who cannot come to their follow-up clinic visits during the 1 year
- Participants who have started taking a smoking cessation medication within 1 year before the registration
- Participants who plan to use any smoking cessation aids and participate in any kind of smoking cessation activities (not limited to smoking cessation therapy) outside the trial
- Participants whose investigator or clinical trial doctor judged them to be unsuitable for participation in this trial owing to other reasons

On the other hand, participants from the control app group will be able to perform only 6 basic functions in their app as follows: (1) show the user guide (how to use the app); (2) enter their profile and set the start date of smoking cessation; (3) display the course of 5 visits during the 12-week treatment with a summary of objectives of each visit; (4) show the date of next appointment; (5) get the contact form for technical support; and (6) display an app version, privacy policy, and other administrative information. The other functions of the CASC app will not be incorporated in the control app. The control app will not include the mobile CO checker either. Note that exhaled CO concentrations will be measured by the medical staff at each clinic visit regardless of the study groups, and these CO data at the clinic will be used in the analysis of study outcomes.

The CASC, control app, and mobile CO checker delivered by CureApp Inc. (Tokyo, Japan) should be carefully managed to ensure they are not used outside the clinical trial at each medical institution. A prescription code will be required to activate the investigational device, and the control group app will be issued by sponsors specific to each practicing medical institution; these sponsors regularly (at least once a year) perform inventory confirming there will be no usage of the app outside the trial.

### Overview of the “CureApp Smoking Cessation” Smartphone app

The CASC smartphone app was developed at CureApp Inc. supervised by the Division of Pulmonary Medicine, Department of Medicine, Keio University School of Medicine. The app runs on both iOS and Android smartphones. Primary physicians will provide the app prescription code to participants at their outpatient clinics. Participants will download the app through their smartphones, activate the app entering the code, and enter their baseline data including age, sex, years of smoking, number of cigarettes per day, prescribed medications (nicotine patch or varenicline), and motivation and self-confidence regarding smoking cessation. These data will be securely sent to the cloud storage, and the AI of our system will assemble tailor-made smoking cessation programs suitable to each participant based on their personal information. Personal data stored on the cloud can be reviewed by primary physicians. The CASC app has the following 4 main components to maximize the therapeutic effects of pharmacological therapy and counseling provided by health care professionals: (1) keeping a smoking cessation diary (once a day); (2) lectures and educational videos helping its users quit smoking; (3) interactive counseling by chat-bot (AI nurse); and (4) daily measurement and recording of exhaled CO concentration levels at home through a mobile CO checker.

Regarding a Web-based personal computer software for primary doctors, it provides a data management app from patients’ CASC mobile apps and advice for physicians to follow the national clinical guidelines appropriately.

### Outcomes

The primary outcome of this study will be the biochemically validated CAR from weeks 9-24. The success of smoking cessation has been defined as (1) self-reported continuous abstinence from weeks 9-24 and (2) exhaled CO concentration no more than 10 ppm at both weeks 12 and 24. In current smoking cessation programs, the CAR from weeks 13-24 drastically decreases, whereas our investigational device is expected to prevent this reduction by covering “treatment gaps” to promote the retention of appropriate recognition and behavioral changes. A single-arm prospective pilot study showed that a CASC app, even without a mobile CO checker, had a significantly higher CAR from weeks 9-24 than the historical cohort that had received standard smoking cessation program [16,17].

In addition, we will evaluate the following secondary outcomes: (1) 7-day point prevalence abstinence at weeks 4, 8, 12, 24, and 52; (2) the CAR from weeks 9-12 and from weeks 9-52; (3) changes in several scores related to smoking cessation, including the Mood and Physical Symptoms Scale (MPSS) [18], 12-item French version of the Tobacco Craving Questionnaire translated into Japanese (FTCQ-12) [19], Kano Test for Social Nicotine Dependence (KTSND) [20], and time to first lapse after initial visit; (4) usage rates of smartphone apps; and (5) the presence of product problems or adverse events.

### Follow-Up Schedule

Table 2 shows the overall follow-up schedule of this trial. At registration, patients’ background profile, exhaled CO concentration, nicotine dependence, and smoking status will be assessed using instruments such as the Tobacco Dependence Screener [21], Brinkman index (multiplied by cigarettes per day and years of smoking), Fagerström Test for Nicotine Dependence (FTND), FTCQ-12, KTSND, and MPSS. The background profile includes age, sex, body weight, years of smoking, cigarettes per day, and medical history at the initiation of device use. Regular follow-up visits will be scheduled at 2 weeks (±7 days), 4 weeks (±7 days), 8 weeks (±14 days), 12 weeks (±14 days), 24 weeks (±28 days), and 52 weeks (±28 days); during these follow-up visits, we will examine patients’ scores regarding FTCQ-12, KTSND (after 8 weeks), and MPSS, as well as exhaled CO concentration, status of nonsmoking, status of device use, and adverse events. Adverse events and concomitant pharmacological therapy will be recorded through the trial.

An independent monitoring staff will conduct on-site data monitoring. In addition, the staff will review trial database to confirm whether principal and subinvestigators at each clinic perform the clinical trial according to the research proposal and standard operation procedures, and data queries will be asked if necessary.

### Participant Withdrawal Criteria

Participants will be discontinued from follow-up visits for this trial when they meet either of the following criteria: (1) participants request discontinuation; (2) researchers consider discontinuation is necessary owing to severe adverse effects, for example; (3) the eligibility criteria was violated; (4) significant deviation from the protocol occurred; (5) study institution terminated trial participation because of good clinical practice violation, for example; (6) participants’ further follow-up visits are considered very difficult to accomplish; (7) continuing smoking even at the 9^th^ week or participant no longer intends to continue the smoking cessation program; (8) sponsor cannot continue the trial; and (9) any other cause in which researchers decide discontinuation is appropriate.

### Sample Size

The primary endpoint will be the CAR from weeks 9-24. We estimated conservatively 52.8% in the CASC treatment group and 40.8% in the control group as the primary endpoint (CARs from weeks 9-24) based on the survey of smoking cessation [22]. Regarding the effect size, we referred data from a clinical trial of maintenance therapy with varenicline [7], which indicated that 24 weeks of varenicline therapy showed 12% of additional abstinence rate than placebo in the total population. Thus, we estimated that 24 weeks of the CASC intervention could obtain additional effect of 12% on abstinence rate compared with the control group. A sample size of 290 in each treatment group will have 80% power using a chi-square test with a 2-sided significance level of .05 (nQuery Advanced 8.2.1).

### Statistical Analysis

All statistical analyses will be performed according to the intention-to-treat principle and using 2-sided at a .05 significance level. The baseline characteristics will be described by the mean and SD, median and quantiles (continuous variables), or proportion (categorical variables). We will examine the outcomes using the full analysis set. For the primary outcome, we will compare CARs from weeks 9-24 comparing the CASC treatment group and the control group using a logistic regression model adjusted for prescribed smoking cessation medications. In addition, secondary outcomes at each defined period will be evaluated with logistic regression analysis adjusted for appropriate covariates between the groups. We will provide descriptive statistics, odds ratios, 95% CIs, and *P* values for each outcome. A statistical analysis plan detailing all statistical computations will be completed prior to the lock of the database. SAS statistical software, version 9.4 or upper version (SAS Institute Inc), will be used for all the analyses.

## Results

A total of 580 participants with nicotine dependence will be recruited from October 2017 to September 2018 or until recruitment is complete. The final 52-week follow-up will be completed in October 2019. We expect that all trial results will be available by the end of 2019.

## Discussion

This is the first randomized controlled trial to evaluate the long-term efficacy of CASC, a smoking cessation support app for participants, which also includes a Web-based software for doctors and a mobile CO checker. We expect that participants in the CASC treatment group, in addition to a 12-week standard smoking cessation program, will exhibit a significantly higher CAR from weeks 9-24 than participants in the control app group.

In recent years, several medical apps for smartphones have shown better clinical outcomes compared with conventional treatment [10-12]. These smartphone apps have also been approved by the US Food and Drug Administration being used in daily clinical practice. In terms of smoking cessation, individuals who used CASC smartphone app without a mobile CO checker achieved 63% of the CAR from weeks 9­-24 compared with those who did not use the app (historical control) [15]. Clickotine, another smartphone app for smoking cessation, also reached 30% of the abstinence rate at 8 weeks [23]. However, it remains unclear whether CASC including a mobile CO checker (which provides exhaled CO concentrations for the user), evidence-based behavioral support, education, and counseling programs for smoking cessation is effective for maintaining long-term abstinence rates in patients with nicotine dependence.

We developed and used a mobile Internet of Things (IoT) device to measure an exhaled CO concentration level in this trial. A level of exhaled CO concentration is a useful biomarker for patients with nicotine dependence [5]. It helps them understand how much harmful CO is accumulated in their body and how steadily the harm is decreasing after smoking cessation. They might feel guilty for a high exhaled CO concentration level and try to start quitting, and in another case, they could be encouraged to keep quitting by seeing the decline of their CO level [24]. These experiences could continuously motivate them and improve adherence to the standard smoking cessation program [25].

There are several benefits to including smartphone apps with an IoT device to clinical settings. First, a mobile app covers treatment gaps between the outpatient clinic visits. It is beneficial to continuously contact participants and keep providing effective treatment programs, which could be one of the most important points for the program success [10,13]. Second, medical IoT devices enable us to easily gather biometric information remotely. Patients no longer need to access their outpatient clinics for testing; this might improve patients’ total adherence to the treatment program and could contribute to construct and promote “telemedicine.” Third, the total development costs of the app are much lower than that of medical drugs or medical devices. These apps are highly cost-effective and might reduce burgeoning medical costs [3].

Improving nicotine dependence treatment programs with therapeutic smartphone apps and IoT devices is important in terms of preventing chronic obstructive pulmonary disease, cardio- and cerebrovascular diseases, and malignant tumors. In addition, these mobile apps and devices can contribute to preventing secondhand smoke, which kills approximately 15,000 people every year in Japan [3]. In addition, our device can be a pioneer of the stand-alone programmed medical device with better treatment outcomes. Improving medical outcomes with mobile apps and devices is challenging; however, it can become a highly cost-effective treatment option in the near future. We believe that CASC, which is a combination of a smartphone app, a Web-based patient management software, and a mobile CO checker, will be able to improve the CAR, decrease smoking prevalence and passive smoking, and reduce future total medical costs, while preventing smoking-related diseases in the world.

## Abbreviations

AI: artificial intelligence
CAR: continuous abstinence rate
CASC: CureApp Smoking Cessation
CO: carbon monoxide
FTCQ-12: 12-item French version of the Tobacco Craving Questionnaire
IoT: Internet of Things
KTSND: Kano Test for Social Nicotine Dependence
MPSS: Mood and Physical Symptoms Scale
